# Wheat Bread Fortification by Grape Pomace Powder: Nutritional, Technological, Antioxidant, and Sensory Properties

**DOI:** 10.3390/foods10010075

**Published:** 2021-01-02

**Authors:** Roberta Tolve, Barbara Simonato, Giada Rainero, Federico Bianchi, Corrado Rizzi, Mariasole Cervini, Gianluca Giuberti

**Affiliations:** 1 Department of Biotechnology, University of Verona, Strada Le Grazie 15, 37134 Verona, Italy; roberta.tolve@univr.it (R.T.); giada.rainero@univr.it (G.R.); federico.bianchi_02@univr.it (F.B.); corrado.rizzi@univr.it (C.R.); 2 Department for Sustainable Food Process, Università Cattolica del Sacro Cuore, via Emilia Parmense 84, 29122 Piacenza, Italy; mariasole.cervini@univr.it (M.C.); gianluca.giuberti@unicatt.it (G.G.)

**Keywords:** bread fortification, grape pomace, agro-industrial by-products, antioxidant activity, phenolic compounds, sensory analysis

## Abstract

Grape pomace powder (GPP), a by-product from the winemaking process, was used to substitute flour for wheat bread fortification within 0, 5, and 10 g/100 g. Rheological properties of control and fortified doughs, along with physicochemical and nutritional characteristics, antioxidant activity, and the sensory analysis of the obtained bread were considered. The GPP addition influenced the doughs’ rheological properties by generating more tenacious and less extensible products. Concerning bread, pH values and volume of fortified products decreased as the GPP inclusion level increased in the recipe. Total phenolic compounds and the antioxidant capacity of bread samples, evaluated by FRAP (ferric reducing ability of plasma) and ABTS (2,2′-azino-bis (3-ethylbenzothiazoline-6-sulfonic acid)) assays, increased with GPP addition. Moreover, the GPP inclusion level raised the total dietary fiber content of bread. Regarding sensory evaluation, GPP fortification had a major impact on the acidity, the global flavor, the astringency, and the wine smell of bread samples without affecting the overall bread acceptability. The current results suggest that GPP could be an attractive ingredient used to obtain fortified bread, as it is a source of fiber and polyphenols with potentially positive effects on human health.

## 1. Introduction

White wheat bread is a worldwide staple food, rich in complex carbohydrates (i.e., starch) and generally poor in dietary fiber and other micro-and macronutrients [1]. However, consumer demand for healthy and high-nutritional-value foods is increasing, and this phenomenon has attracted the attention of food manufacturers. 

Specifically, the UN has set new challenges that must be achieved by the human population. Among the 17 extremely important goals of Agenda 2030, “Responsible consumption and production” and “Health and well-being” are reported. Particularly, the division between economic growth and environmental depletion, the enhancing of resource efficiency, and the promotion of sustainable lifestyles should be the starting point of ecofriendly consumption and production [2].

Grape pomace (GP), the main residue from the winemaking process, represents a promising by-product. It has been evaluated that 17 kg of GP is discarded for about every hectoliter of wine produced [3]. So, GP, in terms of a sustainable economy, is a putative ingredient to reduce industrial waste and to promote economic profit. In addition, GP contains several bioactive compounds, such as polyphenols and dietary fibers, known for their healthy properties [4,5]. Several epidemiological studies on human health have underlined the beneficial role of phenolic compounds in the prevention of several diseases [6,7,8]. In addition, a greater consumption of dietary fiber could reduce the risk associated with the incidence of certain forms of cancer and the development of diabetes. Additionally, dietary fiber improves the bowel transit of feces and the feeling of satiety, reduces blood cholesterol levels, and prevents obesity [9,10,11,12]. However, the recommended intakes (25–30 g/day) are rarely reached by consumers, and market availability of fiber-enriched or fortified foods can help them to achieve the correct daily intake [13].

From this point of view, white wheat bread could be a perfect target for GP fortification [14,15,16,17,18,19]. In this context, it is well known that the addition of new ingredients in white bread formulations generally leads to various changes in technological and nutritional properties [20,21,22]. 

In a framework of developing innovative wheat-based bread, it is therefore essential to assess both the nutritional and technological effect of emerging unconventional ingredients following breadmaking. Then, innovative fortified food products must be subjected to a sensory evaluation to verify their acceptability and to assess how the inclusion of a specific ingredient could modify the sensory profile of the final food product [23].

In a previous study, Hayta et al. [24] concluded that GP powder inclusion significantly contributed to the improvement of bread functional properties, evaluating the antiradical activity, total phenolic content, physicochemical, textural, and sensory properties of the obtained product. In fact, in a framework of developing innovative wheat-based bread, it is essential to assess both the nutritional and technological effect of emerging unconventional ingredients following breadmaking. Then, innovative fortified food products must be subjected to a sensory evaluation to verify their acceptability and to assess how the inclusion of a specific ingredient could modify the sensory profile of the final food product [23]. However, the inclusion of an ingredient rich in fiber could also strengthen the structure of bread doughs, reducing the doughs’ extensibility and affecting the bread volume and texture [25]. Therefore, this study, in addition to evaluating the effect of GP inclusion on dough rheological properties, dealt with the investigation of technological, sensory, and nutritional properties of bread fortified with increasing levels of GP.

## 2. Materials and Methods

### 2.1. Ingredients and Breadmaking

Common white wheat flour was kindly supplied by Macinazione Lendinara SpA (Arcole, Italy). The wheat flour label detailed the following composition: fat 1.2 g/100 g, total carbohydrates 71 g/100 g, protein 11 g/100 g, and dietary fiber 2.3 g/100 g.

Grape pomace (Vitis vinifera cv. Corvina) was kindly provided by Cantina Ripa Della Volta (Verona, Italy). After alcoholic fermentation, GP was pressed and dried in a vacuum oven (VD 115 Binder GmbH, Tuttlingen, Germany; 40 °C, 30 kPa) until the final moisture content of 11.0 g water/100 g dry matter (DM) was reached. The dried pomaces, without grape seeds, stems, and stalks, were ground (GM200 Retsch, Haan, Germany) to a particle size of <200 μm. The GP powder (GPP) was preserved in vacuum packaging at room temperature until analyzed or used for bread preparation as described by Cisneros-Yupanqui et al. [26]. The chemical composition of the dried GPP was as follows: crude protein: 11.19 ± 0.97 g/100 g DM; total dietary fiber: 52.3 ± 2.1 g/100 g DM; ash: 4.17 ± 0.87 g/100 g DM. 

Experimental recipes were obtained by replacing white wheat flour with 0, 5, and 10 g/100 g of GPP, obtaining GP0, GP5, and GP10 bread samples, respectively. The recipe was based on 320 g of composite flours, 210 mL of tap water, 3 g of salt, 15 g of sugar, and 3.5 g of dried brewer’s yeast. The breads were prepared with a commercial breadmaking machine (Panexpress 750 Metal, model 0132/00—Ariete, Italy). Doughs were mixed, fermented for 39 min at 28 °C, and then baked at 170 °C for 65 min. For each formulation, three different doughs (i.e., batches replicates) were made on the same day.

### 2.2. Dough Characterization

A Brabender Farinograph (Brabender, Duisburg, Germany) was used to evaluate the dough mixing properties (AACC method 54-21.02) [27]. Dough water absorption, stability, development time, degree of softening (12 min after maximum), and quality number were considered. 

The viscoelastic behavior of the doughs was investigated using an alveograph (Chopin Technologies, Villeneuve La Garenne, France) (AACC method 54-30) [27]. The parameters recorded were deformation energy (W), tenacity (P), dough extensibility (L), swelling index (G), and the curve configuration ratio (P/L ratio).

Start of gelatinization (°C), gelatinization maximum (AU), and gelatinization temperature (°C) were analyzed using an amylograph (Brabender, Duisburg, Germany) (AACC method 22-10) [27].

### 2.3. Bread Quality Characteristics

#### 2.3.1. Water Activity, Moisture Content, Volume, Specific Volume, and Baking-Loss

The water activity (aw) of bread samples was measured with a Hygropalm HC2-AW-meter (Rotronic Italia, Milano, Italy) at 23 °C, whereas the moisture content was measured by the AACC method 44-15A [27]. The specific volume of the loaves (cm^3^/g) was determined by seed displacement (AACC method 10-05.01) [27] for volume quantification (cm^3^) and weight of samples (g). The baking-loss was determined as the differences in mass between the dough and the baked loaves.

#### 2.3.2. Texture Attributes of Bread Crumb

The texture attributes in terms of firmness of bread crumb were evaluated using a texture analyzer (TVT-6700; Perten Instruments, Stockholm, Sweden) according to the AIB standard procedure for bread firmness measurement [28]. The maximum peak force of compression as bread firmness (N) was measured by a metal cylinder probe (25 mm diameter). For each treatment, five measurements were done for each batch.

#### 2.3.3. Proximate Composition of Breads

Dry matter (DM; method 930.15), ash (method 942.05), crude protein (method 976.05), crude lipid (method 954.02 without acid hydrolysis), total starch (method 996.11, using thermostable α-amylase (Megazyme cat. no. E-BSTAA) and amyloglucosidase (Megazyme cat. no. E-AMGDF)), and total dietary fiber (method 991.43) were considered [29]. Free sugars were assessed using the Megazyme assay kit K-SUFRG 06/14 (Megazyme, Wicklow, Ireland). Batches were analyzed in triplicate.

#### 2.3.4. Color Analysis

The color was measured by a reflectance colorimeter (illuminant D65) (Minolta Chroma meter CR-300, Osaka, Japan) based on the color system CIE – L* a* b*. Analyses were performed at five different points within the crumb and the crust area. Minolta Equations (1) and (2) were used to calculate the total color difference (∆E):

(1) $$\Delta E=\sqrt{{\Delta L}^{2}+{\Delta a}^{2}+{\Delta b}^{2\;}}$$

Δ*L* = (*L* − *L*_0_); ∆*a* = (*a* − *a*_0_); ∆*b* = (*b* − *b*_0_) (2)

where *L*, *a*, and *b* are the measured values of the bread fortified with grape pomace, and *L*_0_, *a*_0_, and *b*_0_ are the values of the control bread.

#### 2.3.5. Determination of Total Phenolic Compounds (TPC), FRAP, and ABTS Assays

The extraction was carried out by stirring 1 g of bread sample with 15 mL of MeOH:HCl 97:3 (*v*/*v*) for 16 h in the dark at room temperature [30]. Supernatants were collected after centrifugation (3500× *g* for 10 min) and used for TPC, ABTS (2,2′-azino-bis (3-ethylbenzothiazoline-6-sulfonic acid)), and FRAP (ferric reducing ability of plasma) radical scavenging activities determination. TPC determination was performed as described by Simonato et al. [31]. Two hundred microliters of extracts were mixed at room temperature with the same amount of Folin–Ciocalteau reagent. After 5 min, 4 mL of Na_2_CO_3_ (0.7 M) and 5.6 mL of Milli-Q water were added. The absorbance was measured at 750 nm (ATi Unicam UV2, Akribis Scientific, Cambridge, UK) after 1 h under stirring in the dark. The TPC is expressed as milligrams of gallic acid equivalent (GAE) per gram of dry matter (DM).

The FRAP solution was prepared by mixing a sodium acetate buffer (300 mM, pH 3.6), a FeCl_3_∙6H_2_O solution (20 mM), and 10 mM of TPTZ solution in HCl 40 mM in a volume ratio of 10:1:1. Then, 1.8 mL of FRAP reagent and 1 mL of Milli-Q water were mixed with 10 μL of the methanolic extract. The absorbance was measured at 593 nm after 10 min at 37 °C [32]. Quantification is expressed as micromolars of Trolox equivalent (TE) per gram of DM.

The ABTS assay was performed starting from a stock solution of the radical cation ABTS + obtained mixing 7 mM ABTS and 2.45 mM K_2_S_2_O_8_ (1:1 ratio) for 16 h at room temperature in the dark. The stock solution was brought at an absorbance of 0.72 ± 0.2 at 734 nm by dilution in Milli-Q water. ABTS + diluted solution (9.8 mL) was mixed with 0.2 mL of the methanolic extract and stirred for 30 min. Absorbance was measured at 734 nm and the results are expressed as micromolars of Trolox equivalent (TE) per gram of DM [33]. 

### 2.4. Sensory Evaluation of Breads

According to Quantitative Descriptive sensory Analysis (QDA), the sensory profile of samples was analyzed as proposed by Vilanova et al. [34]. A trained sensory panel of 16 persons (10 females and 6 males) aged between 22 and 33 years, recruited from the staff and students of the Department of Biotechnology of the University of Verona, was involved. Panelists generated 18 sensory terms and were trained to recognize their intensities. Color uniformity, porosity, crust thickness, fragrance, wine taste, yeast taste, global flavor, sweetness, saltiness, acidity, bitterness, moisture of the crumb, crust hardness, adhesiveness, grittiness, and astringency were considered as sensory attributes and evaluated using a hedonic 9-point scale, where 1 and 9 indicate the lowest and the highest intensity, respectively. For the sensory evaluation, samples were cut into 2 cm thick slices of about 10 g for each sample (including crumb and crust) and placed on a covered plate. All the coded samples were presented in a completely randomized and balanced order. Panelists also commented on the overall acceptability: mean scores above 5 were considered acceptable (neither like nor dislike).

### 2.5. Statistical Analysis

All data reported (i.e., mean values ± standard deviation) represent the means of at least three measurements. Analysis of variance (ANOVA), with a post hoc Tukey test at *p* < 0.05, was used for mean comparison. Statistical analyses were performed using the software XLSTAT Premium Version (2019.4.2, Addinsoft SARL, Paris, France).

## 3. Results and Discussion

### 3.1. Effect of Grape Pomace inclusion on Dough Rheological Properties

The addition of GPP significantly influenced the dough technological properties (Table 1). The amount of water absorbed by the doughs increased with the increasing levels of GPP in the recipe, ranging from 55.50% to 60.03% for GP0 and GP10 dough, respectively (*p* < 0.05). This increase is related to the higher dietary fiber content of the dough after GPP addition, as observed by Mironeasa [35]. Further, dietary fiber is characterized by a high number of hydroxyl groups which allow greater interactions with water molecules through hydrogen bonds [36]. The dough development time and its stability are suitable indicators of flour firmness, with higher values indicating a firmer dough. Compared to GP0, both GP5 and GP10 doughs showed no change in the development time (being on average 1.39 min; *p* > 0.05), while an increase in the stability was observed, ranging from 5.80 to 8.27, for GP0 and GP10, respectively (*p* < 0.05). Theoretically, the reduction in wheat gluten proteins caused by the GPP inclusion would lead to a decrease in the dough strength. In this case, the strength may have been enhanced by phenolic compounds of GPP, in line with previous findings [37]. In particular, the condensed tannins of the GPP can interact mainly with the glutenin fractions of wheat flour through hydrogen bonds and hydrophobic interactions. Due to their longer and broader conformation, condensed tannins have better access to the glutenin structure for noncovalent interactions with amino acid residues compared with the globular gliadins [38]. A higher content of phenolic substances in fortified dough samples could explain the reduction of the degree of softening [37], defined as the difference between the value recorded at the peak and the value recorded after 12 min. Moreover, quality numbers of GP5 and GP10 sharply increased compared with GP0, indicating a gain of flour ability in the production of doughs resistant to mechanical stress, as seen by Davoudi et al. [39].

The alveograph results are presented in Table 2. The extensibility value (L) reveals the ability of the dough to expand without breakdown, and it decreased following GPP addition. This could be explained by the high fiber content of the GPP, which could compete with the protein gluten for water absorption, forming a weaker gluten network, thus resulting in lower extensibility [40]. The tenacity of the dough (P), which indicates the gas-retaining ability of the dough, increased with the inclusion of GPP, ranging from 76.67 to 179.33 mm for GP0 and GP10, respectively, (*p* < 0.05). This can be related to the presence of stronger interactions between polysaccharides and the gluten proteins [40]. The P/L ratio is an index used for the gluten behavior. The addition of GPP significantly increased the P/L of the doughs. This could be caused by a strong interaction between cellulose contained in the fiber fraction and the flour protein, as already reported by Fendri et al. [41]. In particular, the P/L values were always above the range recommended for bakery leavened products, which should not be higher than 2 [42]. The G value (the size of bubbles after air insufflation) was significantly higher in GP0 dough than GP5 and GP10, whereas the deformation energy (W), described as the area under the curve of the alveogram, decreased in fortified dough samples due to the higher P and lower L values. Similar results were obtained in doughs containing different amounts of almond skin powder and in dough with barley husk powder [43,44].

The incorporation of GPP significantly affected the gelatinization maximum of the dough (Table 3), defined as the maximum viscosity reached. Viscosity increased in GP5 and GP10 doughs compared with GP0 dough, as observed in doughs enriched with white grape peel flour of different particle sizes [45]. This result could be associated with polymer complexes from mixing derived from interactions between fiber and amylose and low-molecular-weight amylopectin chains [46]. Moreover, starch may have been exposed to a faster gelatinization process, leading to higher water absorption from dietary fiber of GPP [35]. On the contrary, the addition of GPP had no effect on the starting temperature of starch gelatinization and the gelatinization temperature at maximum viscosity.

### 3.2. Physiochemical Characterization of Grape Pomace Powder and Breads

The moisture content, a_w_, and pH values of GPP were respectively 6.17 ± 0.09 g/100 g DM, 0.33 ± 0.01, and 3.39 ± 0.01.

Technological properties and chemical values of breads in terms of water activity, moisture content, pH, volume, and specific volume along with the firmness values are reported in Table 4. The a_w_, moisture content, and baking-loss of GP0, GP5, and GP10 samples showed no statistical differences, in contrast with other research work on bread fortification, where the moisture content generally increased with the degree of fortification [47,48,49]. The pH value decreased significantly in GPP-fortified bread samples compared with the control. Evidently, GPP inclusion lowered the pH in fortified bread samples, according to other researchers [37,50].

**Figure 1:**
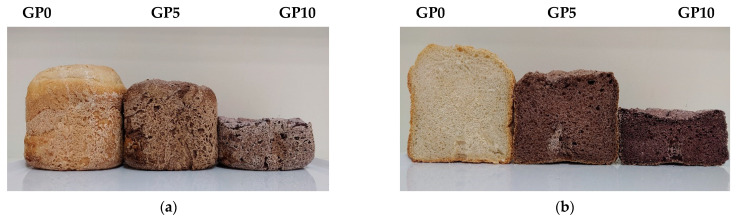
(**a**) Crust side of bread samples; (**b**) crumb side of bread samples. Control bread (GP0) and bread fortified with grape pomace powder (GP5 and GP10).

The leavening capacity and volume of bread depend on several factors such as the kneading condition, leavening time, rheology of the dough, type of ingredients, temperature, and type of yeast used. Inducted acidic conditions and the consequent increase in the number of positive charges in the doughs may have altered the gluten network of the samples, leading to the unfolding of gluten proteins [51]. Such structural changes would lead to a less extensible and more tenacious dough in fortified samples, as seen in alveographic and farinographic results, and finally, to a reduction of volume and specific volume (Figure 1). Moreover, acidic conditions could promote the gluten proteins’ solubilization and, thus, the instability of the gluten network [52]. Indeed, endogenous proteases from wheat flour work better at a low pH and this could intensify gluten proteolysis, especially of GMP (glutenin macropolymer) [51]. 

In particular, *Saccharomyces cerevisiae*, responsible for dough leavening, is reported to have an optimal pH growth rate ranging between 4 and 6. Nevertheless, low pH conditions could provide a more stressful environment, thus reducing the yeast activity [53]. According to Fu et al. [54] and Seczyk et al. [55], the bread volume decreased with the increase of the fortification level. In addition, the substitution of wheat flour with a high-dietary-fiber ingredient such as GPP in bread formulation could have impaired the volume of the samples. In particular, dietary fiber constitutes about 52% of GPP and can subtract water to starch granules and protein networks, affecting the overall capacity of the gluten network to retain gas bubbles [54]. High-dietary-fiber ingredients can also have a negative role on kneading by impairing air inclusion, as well as gaseous release and distribution, with a negative effect on the leavening capacity [56]. Finally, the inhibition of amylase should be also considered. Indeed, polyphenols may interact and inhibit amylase, thus contributing negatively to starch hydrolysis and to the maltose accessibility for yeasts and influencing the volume growth during the leavening stage, as suggested by other researchers [9,37]. 

The firmness of bread crumb increased with the increasing level of GPP, ranging from 1.0 to 23.2 N for GP0 and GP10, respectively (*p* < 0.05). These results appear consistent with those previously reported for dough and bread quality attributes. The addition of GPP may have caused a weak gluten network formation with poor gas retention ability, thus contributing to the hardening effect of bread crumb. The present findings are in agreement with Sui et al. [57], where an increase in hardness was reported in wheat-based bread crumb with increasing levels of an anthocyanin-rich black rice extract powder. Similar results have also been reported in breads formulated with increasing levels of dietary fiber extracted from culinary banana bract [58].

### 3.3. Chemical Composition of Breads

Among the samples, similar crude protein (being on average 12.2 g/100 g DM) and free sugar (being on average 1.8 g/100 g DM) contents were recorded (Table 5). On the contrary, the inclusion of a higher level of GPP caused a decrease in the total starch content (ranging from 85.5 to 75.3 g/100 g DM for GP0 and GP10, respectively; *p* < 0.05) and an increase in the total dietary fiber content (from 2.8 to 6.3 g/100 g DM for GP0 and GP10, respectively; *p* < 0.05). The different chemical compositions of the selected ingredients, as well as their inclusion level, can explain the present findings. Similar results have already been reported in baked foods containing GPP at different inclusion levels [50]. In addition, from a nutritional standpoint, the GP10 bread can be considered a food product high in dietary fiber, having a total dietary fiber content higher than 6 g/100 g. Higher total dietary fiber intake can prevent certain chronic diseases such as diabetes, obesity, and hypertension [59].

### 3.4. Color Analysis

The change in color of crumb and crust of breads fortified with increasing levels of GPP is summarized in Table 6. In the control bread (GP0), the crust color was darker than the crumb due to different Maillard and caramelization reactions [60]. The GPP inclusion reversed this trend because of the GPP-darkened color parameters. According to Hayta et al. [24] and Nakov et al. [50], GPP inclusion caused a significant reduction in brightness (L*) in samples GP5 and GP10, both in the crumb and the crust. As expected, an increase in the a* parameter was evident according to the GPP increment in the bread crumb, while an opposite trend was observed in the crust. This could be due to the fact that anthocyanins, responsible for the red GPP pigmentation, are less degraded in the crumb since it undergoes lower heat treatment and maintains a higher moisture level than the crust [61]. Finally, a significant decrease in the b* parameter was observed with the progressive supplement of GPP in both crumb and crust. The total color difference (∆E) is generally used to describe the color variation. The ∆E values revealed that GP5 and GP10 led to high color variation as the concentration of added GPP increased. This trend was observed both in the crumb and the crust but was less pronounced in the latter.

### 3.5. Polyphenols and Antioxidant Activity

In the present study, the total polyphenol content (TPC) and antioxidant activity of GPP and fortified bread samples were tested. GPP achieved a TPC value of 15.02 ± 0.63 mg GAE/g DM, while the antioxidant activity, assessed by FRAP and ABTS, resulted in 248.74 ± 9.53 µM TE/g DM and 213.53 ± 10.16 µM TE/g DM, respectively.

The TPC and both antioxidant tests increased significantly in fortified bread GP5 and GP10 compared with the control bread GP0, with high correlation coefficients between them (r = 0.999 CPT vs. FRAP and r = 0.979 CPT vs. ABTS) (Table 7). The TPC increased 3.5-fold and 7-fold as GPP replacement increased from 0% to 5% and from 0% to 10%, respectively. Slightly lower increases were observed by Hayta et al. [24], with 1.9-fold and 2.5-fold for the same GPP inclusion levels reported in this study. However, Hoye and Ross [62], with a substitution of 10% wheat flour by grape seed flour, reported a 20-fold increase. These discrepancies could be explained by the grape variety and the presence/absence of grapeseed flour in the dried GP [37].

### 3.6. Sensory Evaluation

**Figure 2:**
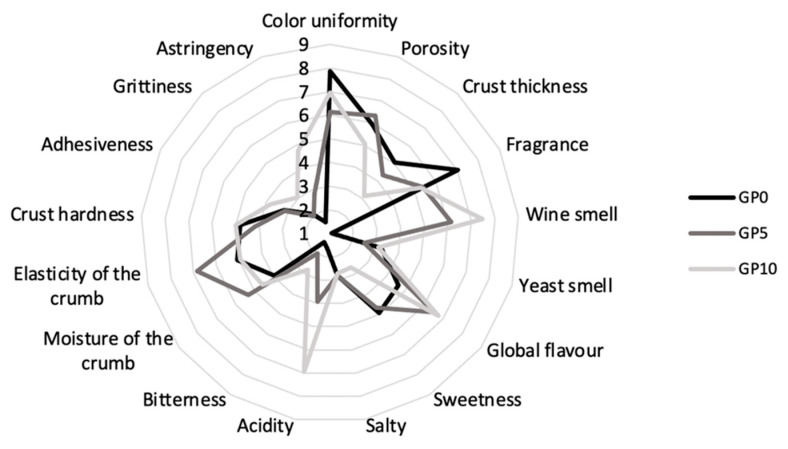
Sensory profile of quality attributes of bread fortified with different grape pomace levels (GP0 black line; GP5 gray line; GP10 light gray line). Sensory attributes were evaluated using a hedonic 9-point scale, where 1 and 9 indicate the lowest and the highest intensity, respectively.

Substitution of wheat by GPP significantly influenced most of the selected sensory attributes (Figure 2). In terms of appearance, GPP inclusion significantly affected color uniformity, porosity, and crust thickness. In particular, crust thickness decreased as the fortification level increased. As for the aroma, increasing the amount of GPP in the dough significantly decreased the overall intensity of fragrance, defined as the characteristic bread scent. Instead, as expected, the wine smell significantly increased as the GPP inclusion increased. GPP inclusion also significantly affected the taste and flavor of the bread: in particular, the global flavor and acidity increased significantly with the GPP inclusion, while the sweet taste was reduced by the GPP inclusion. In terms of texture and tactile sensations, GPP fortification significantly influenced the crumb moisture, the crust hardness, the grittiness, and the astringency. Finally, the GPP addition did not have a significant impact on the overall acceptability of the product. Indeed, the overall acceptability recorded was 6.53 ± 1.62 for GP0 bread, 6.65 ± 1.69 for GP5, and 5.59 ± 2.12 for GP10. In all the samples, the threshold value of 5 was exceeded. Likewise, Walker et al. [63] reported that bread fortified with 10% of pinot noir grape pomace was acceptable by consumers, while an inclusion of 5% was the highest fortification level for bread made with grape seed flour [62].

## 4. Conclusions

The outcomes of our research show that GPP inclusion influences the technological, nutritional, and organoleptic properties of both dough and bread. GPP addition improved water absorption and quality number and reduced the softening degree of doughs without affecting the time and temperature of gelatinization. Moreover, GPP increased the tenacity and P/L ratio but lowered the extensibility, G value, deformation energy (W), specific volume, and pH of bread samples. GPP inclusion modified the chemical composition of bread along with the color parameters. As expected, incremental addition of GPP resulted in a significantly higher amount of TPC in bread samples and allowed increasing antioxidant activity in GP5 and GP10 compared with the control. Finally, although GPP inclusion significantly influenced aroma, taste, appearance, and flavor, a nonsignificant impact on the overall acceptability of the fortified products was observed. In conclusion, GPP represents a suitable ingredient for bakery purposes since it has a significant impact on total dietary fiber as well as on the polyphenol content and antioxidant activity of the fortified bread, achieving a similar acceptability score compared to traditional bread.

## Figures and Tables

**Table 1:** Farinograph characteristics of dough supplemented with grape pomace powder.

Sample	Water Absorption (%)	Stability (min)	Development Time (min)	Degree of Softening (UB)	Quality Number
**GP0**	55.50 ± 0.00 ^a^	5.80 ± 0.72 ^a^	1.40 ± 0.10 ^a^	76.33 ± 4.73 ^a,b^	41.67 ± 13.05 ^a^
**GP5**	56.80 ± 0.17 ^b^	7.50 ± 0.00 ^b^	1.37 ± 0.12 ^a^	81.33 ± 6.03 ^a^	80.33 ± 3.06 ^b^
**GP10**	60.03 ± 0.46 ^c^	8.27 ± 0.55 ^b^	1.40 ± 0.10 ^a^	67.00 ± 3.00 ^b^	87.33 ± 6.51 ^b^

Data with different letters in each column are significantly different for *p* < 0.05.

**Table 2:** Alveograph characteristics of dough supplemented with grape pomace powder.

Sample	L (mm)	P (mm)	P/L	G (cm^3^)	W (10^−4^ J)
**GP0**	106.33 ± 11.72 ^a^	76.67 ± 4.51 ^a^	0.73 ± 0.12 ^a^	22.97 ± 1.29 ^a^	268.67 ± 8.62 ^a^
**GP5**	30.00 ± 1.00 ^b^	165.33 ± 4.62 ^b^	5.52 ± 0.32 ^b^	12.20 ± 0.20 ^b^	222.67 ± 3.51 ^b^
**GP10**	15.33 ± 1.15 ^c^	179.33 ± 9.02 ^b^	11.75 ± 1.12 ^c^	8.70 ± 0.35 ^c^	118.33 ± 11.72 ^c^

Data with different letters in each column are significantly different for *p* < 0.05.

**Table 3:** Amylograph characteristics of dough supplemented with grape pomace powder.

Sample	Gelatinization Maximum (AU)	Start of Gelatinization (°C)	Gelatinization Temperature (°C)
**GP0**	1301.67 ± 17.56 ^a^	64.00 ± 0.87 ^a^	92.30 ± 0.75 ^a^
**GP5**	1831.67 ± 42.52 ^b^	64.00 ± 0.87 ^a^	93.30 ± 0.52 ^a^
**GP10**	1943.33 ± 11.55 ^c^	64.50 ± 1.50 ^a^	93.70 ± 0.75 ^a^

Data with different letters in each column are significantly different for *p* < 0.05.

**Table 4:** Water activity, moisture content, pH, volume, and specific volume along with the firmness values and baking-loss of control bread (GP0) and bread fortified with grape pomace powder (GP5 and GP10).

Sample	Water Activity	Moisture content (%)	pH	Volume (cm^3^)	Specific Volume (cm^3^/g)	Firmness (N)	Baking-Loss (%)
**GP0**	0.96 ± 0.01 ^a^	43.47 ± 0.42 ^a^	5.73 ± 0.38 ^a^	2623 ± 52 ^a^	5.52 ± 0.11 ^a^	1.0 ± 0.10 ^a^	13.87 ± 0.00 ^a^
**GP5**	0.97 ± 0.00 ^a^	41.46 ± 1.71 ^a^	4.44 ± 0.03 ^b^	1691 ± 104 ^b^	3.59 ± 0.27 ^b^	21.8 ± 1.44 ^b^	14.51 ± 1.92 ^a^
**GP10**	0.97 ± 0.01 ^a^	40.58 ± 3.70 ^a^	3.98 ± 0.01 ^c^	1334 ± 63 ^c^	2.82 ± 0.13 ^c^	23.2 ± 1.65 ^c^	14.05 ± 0.77 ^a^

Data with different letters in each column are significantly different for *p* < 0.05.

**Table 5:** Chemical composition (g/100 g dry matter) of experimental breads formulated with increasing levels of grape pomace powder (GPP) in the recipe.

Sample	Crude Lipid	Crude Protein	Total Starch	Total Dietary Fiber	Ash	Free Sugars
**GP0**	0.12 ± 0.05 ^a^	12.4 ± 0.05 ^a^	85.5 ± 2.82 ^c^	2.8 ± 1.00 ^a^	1.0 ± 0.01 ^a^	1.6 ± 0.02 ^a^
**GP5**	0.50 ± 0.22 ^b^	12.3 ± 0.13 ^a^	82.9 ± 0.89 ^b^	3.9 ± 0.74 ^b^	1.1 ± 0.01 ^a^	1.8 ± 0.03 ^a^
**GP10**	0.87 ± 0.06 ^b^	12.1 ± 0.03 ^a^	75.3 ± 1.95 ^a^	6.3 ± 1.07 ^c^	1.4 ± 0.02 ^b^	1.9 ± 0.03 ^a^

Data with different letters in each column are significantly different for *p* < 0.05.

**Table 6:** Color analysis of bread samples expressed as L*(lightness), a* (red/green), and b* (blue/yellow) values. ∆E (total color difference)

Sample	Crust			∆E	Crumb			∆E
	L*	a*	b*		L*	a*	b*	
**GP0**	59.92 ± 1.07 ^a^	6.90 ± 0.59 ^a^	27.71 ± 1.13 ^a^	nd	64.78 ± 1.03 ^a^	1.70 ± 0.28 ^a^	28.70 ± 1.41 ^a^	nd
**GP5**	55.47 ± 0.39 ^b^	5.71 ± 0.47 ^b^	13.84 ± 0.28 ^b^	14.61	48.34 ± 0.58 ^b^	4.25 ± 0.56 ^b^	17.45 ± 0.91 ^b^	20.08
**GP10**	52.25 ± 0.65 ^c^	4.62 ± 0.39 ^c^	11.28 ± 0.16 ^c^	18.28	47.43 ± 0.08 ^b^	5.89 ± 0.06 ^c^	14.74 ± 1.16^c^	22.66

Data with different letters in each column are significantly different for *p* < 0.05. nd (not determined).

**Table 7:** Total phenolic compounds (TPC) and antioxidant activity (FRAP and ABTS) of control bread (GP0) and bread fortified with different percentages of grape pomace (GP5 and GP10).

Sample	TPC (mg GAE 100g^−1^ DM)	FRAP (μM TE 100 g^−1^ DM)	ABTS (μM TE 100g^−1^ DM)
**GP0**	29.08 ± 1.45 ^a^	199.72 ± 9.69 ^a^	240.00 ± 7.90 ^a^
**GP5**	101.5 ± 7.68 ^b^	795.26 ± 63.11 ^b^	999.50 ± 24.78 ^b^
**GP10**	207.06 ± 9.25 ^c^	1577.39 ± 87.20 ^c^	1540.83 ± 47.45 ^c^

Values with different superscripts within the same column are significantly different for *p* < 0.01.

## Data Availability

Not applicable.
